# Subjective voice quality, communicative ability and swallowing after definitive radio(chemo)therapy, laryngectomy plus radio(chemo)therapy, or organ conservation surgery plus radio(chemo)therapy for laryngeal and hypopharyngeal cancer

**DOI:** 10.1093/jrr/rru093

**Published:** 2014-10-26

**Authors:** Marcella Szuecs, Thomas Kuhnt, Christoph Punke, Gabriele Witt, Gunther Klautke, Burkhard Kramp, Guido Hildebrandt

**Affiliations:** 1 Department of Radiotherapy, University of Rostock, Südring 75, 18059 Rostock, Germany; 2 Department of Radiotherapy, University of Leipzig, Stephanstraße 9a, 04103 Leipzig, Germany; 3 Department of Otolaryngology, Head and Neck Surgery ‘Otto Körner’, University of Rostock, Doberaner Straße 137–139, 18057 Rostock, Germany; 4 Department of Radiation Oncology, Hospital Chemnitz, Bürgerstraße 2, 09113 Chemnitz, Germany

**Keywords:** radiation, surgery, quality of voice, dysphagia

## Abstract

This retrospective analysis focusses on the impact of therapy on perceived long-term post-cancer treatment function. A validated questionnaire including items and components for the assessment of communicative ability, quality of voice and swallowing was sent to 129 patients. All patients were treated between 1998 and 2007. A total of 76 patients (58.9%) with carcinoma of the larynx or hypopharynx replied to the questionnaire. Data was evaluated retrospectively. Therapy delivered was definitive radio(chemo)therapy (defchRT/RT) (21/76, 28%), laryngectomy + radio(chemo)therapy (LE + chRT/RT) (28/76, 37%), or larynx conservation surgery + radio(chemo)therapy (LCS + chRT/RT) (27/76, 36%). Radiotherapy was administered using 2D- or 3D-conformal planning. The most common concomitant chemotherapy delivered was cisplatin + 5FU. For statistical analyses of the components, averages were calculated and tested using the Kruskal–Wallis test and the U-test of Mann and Whitney. Differences were assessed by the Monte Carlo method or Fisher's exact test. The single item rates were compared with Fisher's exact test. Mean follow-up was 56.7 months (range, 8–130 months). After defchRT/RT, patients trended towards more substantial–strong hoarseness compared with LCS + chRT/RT (*P* = 0.2). After LE, patients were dissatisfied with their artificial larynx/electrolarynx and the tone of their voice (*P* = 0.3, *P* = 0.07) and communicative ability (*P* = 0.005, *P* = 0.008) compared with those treated with defchRT/RT and LCS + chRT/RT, respectively. Dysphagia and additional percutaneous endoscopic gastrostomy (PEG) feeding were more frequent after defchRT/RT in comparison with the other two groups (*P* < 0.05). Voice quality and communicative ability were slightly worse after defchRT/RT and LE + chRT/RT, but satisfying with all treatment modalities. Further development of the therapy approach is necessary to reduce long-term side effects, with measures of post-treatment function as important endpoints.

## INTRODUCTION

Since the first reports of effective larynx preservation protocols of the Veterans Affairs Laryngeal Cancer Study Group in 1991 [1], different therapeutic options for patients with carcinoma of the larynx and hypopharynx have been developed for improving tumour control. Such diseases should therefore be treated exclusively in a multidisciplinary team, with consistent involvement of the patient [2].

Treatment of T1 and T2a tumours of the glottis and supraglottis without lymph node involvement with either surgery or radiotherapy lead to good functional outcomes for speech and swallowing, along with excellent local control [3, 4, 5].

For locally advanced resectable tumours, it is feasible to use a combined therapy approach, consisting of larynx conservation surgery (LCS) and neck dissection with adjuvant radiation/chemoradiation. However, improvement of functional outcome from this type of therapy is uncertain if organ preservation of the larynx is not possible because of local tumour extension, limitations of speech caused by the tumour itself, or a destruction of essential swallowing structures of the larynx caused by the tumour. A laryngectomy (LE) with neck dissection and subsequent radio(chemo)therapy is then preferred [3]. For patients with UICC (Union for International Cancer Control) advanced Stages III or IVA/B who have unimpaired swallowing function, or for those who desire larynx conservation, definitive chemoradiation is feasible as an alternative to surgery [6].

There is also an increasing influence of inductive regimes featuring intensified chemotherapy as a response-adapted effort to decide between a subsequent surgical approach with LE and an organ-preserving approach with chemoradiotherapy [7].

Several studies have evaluated the efficacy of definitive chemoradiotherapy over LE + adjuvant radio(chemo)therapy. These studies indicate equivalent oncologic outcomes with equal survival rates [8–13]. However, intensive concurrent chemoradiation is associated with increased side effects, which might affect quality of voice and swallowing function over a long time [14]. A new or persistent hoarseness six months after radiation/chemoradiation or endoscopic resection of early laryngeal cancer is the most frequent form of voice impairment after treatment [15, 16]. Resulting xerostomia, muscle atrophy, erythema and fibrosis of the larynx are considered to be causal factors of hoarseness after radiation/chemoradiation [14]. Moreover, fibrosis of the oesophagus limits the structural function of the oesophagus and impairs swallowing [14, 17]. A higher-grade xerostomia also impairs the passage of food. In addition, impairment of swallowing correlates with the dosage of radiotherapy delivered to the anatomic structures involved in swallowing, especially the Musculi constrictores pharyngis and the glottic/supraglottic regions of the larynx [18, 19].

Many studies have thoroughly investigated the acute toxicity of radiation or chemoradiation, but useful documentation of the long-term effects of toxicity is only available for relatively few patients [20].

In summary, very little attention has been given to the question of how therapy affects a patient's long-term subjective therapy experience. These failures are recognized in current investigations and taken into account in ongoing clinical trials, but meaningful data are still lacking for a patient's long-term perceived post-cancer treatment function arising from particular individual treatment approaches. Similarly, detailed information about perceived post-treatment swallowing function and communicative ability is needed [9, 13, 21]. Therefore, measures of voice, swallowing function, and quality of life should be important endpoints. A range of measuring tools are used in these studies, including the Voice-Related Quality of Life Measure (V-RQOL), the List Performance Status Scale for Head and Neck Cancer Patients (PSS-HN), the European Organization for Research and Treatment of Cancer Quality of life Questionnaire-C30 (EORTC QLQ-C30) and the head and neck module (EORTC QLQ-H&N35) [9, 21].

The aim of this analysis was to evaluate the patients' experienced impact on post-therapy communication/voice quality and swallowing function after treatment with definitive chRT/RT, LE + chRT/RT, or LCS + chRT/RT. We paid special attention to assessing whether organ preservation is associated with the perception of improved voice and swallowing function, or if the function is perceived as impaired due to increased toxicity after definitive chRT/RT.

## MATERIALS AND METHODS

### Patients

All patients provided informed consent before the analysis commenced, and this retrospective study was approved by the local ethics committee.

Patients with predominantly UICC Stage III and IVA/B tumours of the larynx and hypopharynx, who were curatively treated between January 1998 and December 2007, were included in this retrospective study. Dysphagia and hoarseness at the time of diagnosis were assessed retrospectively from the patients' medical records. Patient characteristics are shown in Table 1.

**Table 1.** Patient characteristics

Characteristics	defchRT/RT	LE + chRT/RT	LCS + chRT/RT	Total
	patients *n* (%)	patients *n* (%)	patients *n* (%)	patients *n* (%)
**Total number**	21 (100)	28 (100)	27 (100)	76 (100)
**Gender**				
Female	4 (19)	1 (4)	3 (11)	8 (11)
Male	17 (81)	27 (96)	24 (89)	68 (89)
**T category**				
T1	4 (19)	0 (0)	5 (19)	9 (12)
T2	0 (0)	6 (21)	11 (41)	17 (22)
T3	9 (43)	12 (43)	9 (33)	30 (39)
T4	8 (38)	10 (36)	2 (7)	20 (26)
**N category**				
N0	9 (43)	13 (46)	10 (37)	32 (42)
N1	2 (9)	6 (21)	4 (15)	12 (16)
N2	9 (43)	9 (32)	13 (48)	31 (41)
N3	1 (5)	0 (0)	0 (0)	1 (1)
**M category**				
M0	21 (100)	28 (100)	27 (100)	76 (100)
M1	0 (0)	0 (0)	0 (0)	0 (0)
**UICC stage**				
I	4 (19)	0 (0)	2 (7)	6 (8)
II	0 (0)	1 (4)	5 (19)	6 (8)
III	4 (19)	10 (36)	7 (26)	21 (28)
IVA	12 (57)	17 (61)	13 (48)	42 (55)
IVB	1 (5)	0 (0)	0 (0)	1 (1)
**Histology**				
Squamous cell carcinoma	21 (100)	26 (93)	25 (93)	72 (95)
other	0 (0)	2 (7)	2 (7)	4 (5)
**Tumour site**				
Hypopharynx	9 (43)	10 (36)	7 (26)	26 (34)
Larynx	12 (57)	18 (64)	20 (74)	50 (66)
**Age at diagnosis**				
Mean (years)	62.6	55.6	59.8	59
Range (years)	41–74	43–70	42–74	41–74

defchRT/RT = definitive radiochemo-/radiotherapy, LE + chRT/RT = laryngectomy + radiochemo-/radiotherapy, LCS + chRT/RT = larynx conservation surgery + radiochemo-/radiotherapy.

### Surgery

#### Larynx conservation surgery and laryngectomy

Based on the site and extent of the tumour, either classic open surgery (via an external neck incision) or endolaryngeal surgery (mostly transorally with laser) as a supraglottic or glottic partial LE (including epiglottectomy, cordectomy, and horizontal partial LE) was performed. Furthermore, tumour resections were performed as transoral laser microsurgeries. In some cases, a hemipharyngectomy or pharyngectomy was performed.

Depending on the site and extent of the tumour, total LE was combined with partial pharyngectomy. For pharyngeal reconstruction, a primary closure was performed. For good voice prosthesis function, a cricopharyngeal myotomy was arranged. Intraoperatively, a placeholder for the voice prosthesis was applied, after which an Eska Herrmann^®^ voice prosthesis was installed at 10–12 days post-operation.

### Radiotherapy

Radiotherapy was performed—either 2D conventional or 3D conformal. Treatment planning was done with a simulator and CT data, according to the guidelines of the International Commission on Radiation Units and Measurements (ICRU) Reports 29, 50 or 62 [22–24]. Radiotherapy was performed with a linear accelerator (Primus®, Siemens, Erlangen, Germany) and a telecobalt source with 6–15-MV photons or electron energies of 6–12 MV. Isocentric field techniques were used. The median single dose delivered was 2.0 Gy (range, 1.8–2.0 Gy) and the median total tumour dose was 64.0 Gy (range, 56.0–76.0 Gy). Some patients were initially irradiated normofractionated and then hyperfractionated- accelerated (HART). The initially delivered single dose was 2.0 Gy and the subsequent twice-a-day (at least 6 h apart) single dose was 1.4 Gy, delivered up to a total cumulative dose of 70.6 Gy (range, 67.6–73.4 Gy). Dose calculation related to the reference point of the planning target volume.

### Chemotherapy

Concurrent chemotherapy was given on radiotherapy Days 1–5 and 29–33 and consisted of either cisplatin alone administered intravenously (at a dose of 20 mg/m² body surface area/day) as a short infusion or in combination with a continuous infusion for 120 h of 5-fluorouracil (5-FU) (800 mg/m² body surface area/day). Due to contraindications to cisplatin, some patients received carboplatin.

A few patients received twice-weekly paclitaxel (20–25 mg/m²/body surface area/day), or etoposide (100 mg/m²/body surface area/day) on radiation Days 1–3 and 29–31, or weekly cisplatin (30 or 35 mg/m² body surface area/day) over the entire irradiation course.

### Therapy groups

A total of 21/76 (28%) patients were treated with defchRT/RT, 28/76 (37%) with LE + chRT/RT, and 27/76 (36%) with LCS + chRT/RT. 67/76 (88%) patients received a primary therapy and 9/76 (12%) a curative-intended recurrence therapy. Normofractionated radiotherapy was delivered to 67/76 (88%) patients, and a hyperfractionated accelerated radiotherapy (HART) to 9/76 (12%) patients. Chemotherapy was administered to 33/50 (66%) patients, with the majority of respondents receiving cisplatin + 5-FU. Patient characteristics are listed in Table 1.

### Questionnaire

**Fig. 1.**
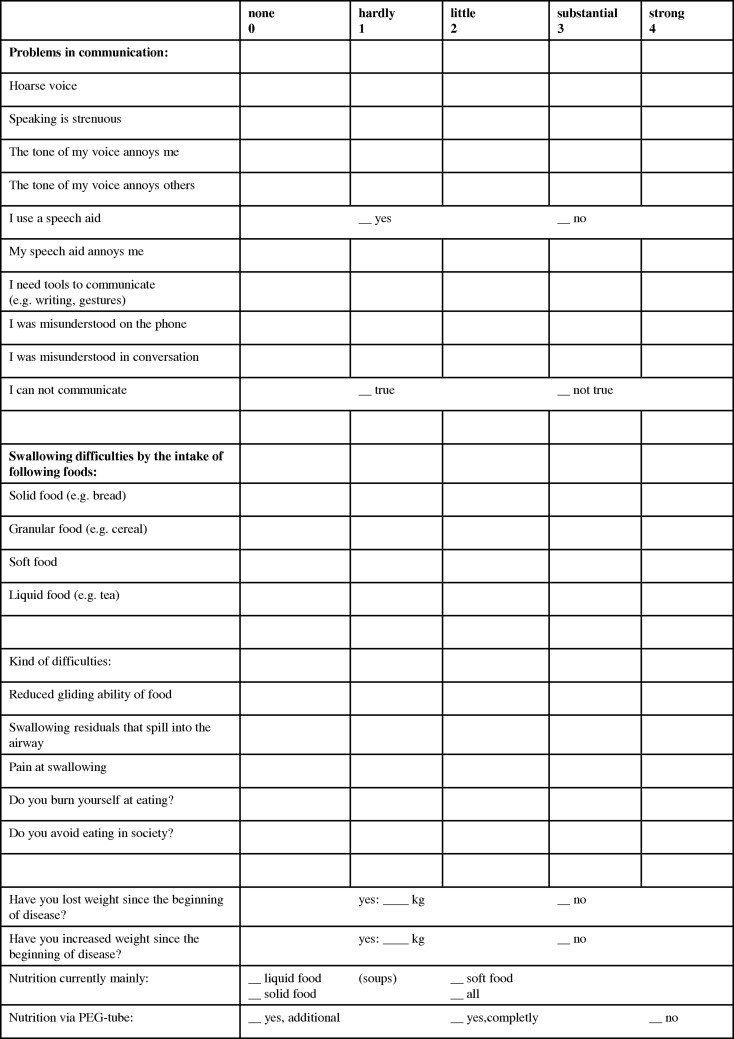
Extract from the questionnaire components communication/voice and swallowing.

All patients who had been alive at the cut-off date received a questionnaire with items and components for the assessment of communicative ability/quality of voice and swallowing to determine their subjective therapy experience. The patients were asked which symptoms occurred in the last few weeks. The questionnaire was developed with reference to the evaluated quality of life questionnaires by Garz [25] and Meuer [26], but augmented with our own additions. A 5-point Likert scale was chosen, with ‘0’ representing no symptoms and ‘4’ representing strong symptoms. The items of the components for communication and swallowing are shown in Fig. 1.

### Statistics

Statistical analysis was performed using SPSS 15.0. Evaluation of the patient data was performed using descriptive statistics. Percentages refer only to those patients who replied to the items of the components. For analysis of the components, communication and swallowing averages were calculated and tested using the Kruskal–Wallis test and the U-test of Mann and Whitney to assess group differences. The Monte Carlo method or Fisher's exact test was used to test the statistical significances of differences between the three defined treatment groups. Tests were 2-tailed. A *P*-value of≤0.05 was considered to be significant. We included all patients who gave complete responses to the items of the components. If a particular question was unanswered too frequently, we did not consider it for the mean-values comparison. Thereafter, each item of the component was assessed using crosstabs and Fisher's exact test. The evaluation date was 1 January 2009. In view of the exploratory nature of the study, no adjustments were made for multiple testing.

## RESULTS

According to the ‘Cancer registry information Mecklenburg–Vorpommern’, 129 head and neck cancer patients were still alive on the date of questionnaire mailing (1 January 2009). The mean follow-up time was 56.7 months (median 52.5 months, range 8–130 months) from completion of the treatment to the mailing of the questionnaire for the total collective. For the defchRT/RT group, the mean follow-up time was 45.3 months (median 27.0 months, range 11–106 months), for the LE + chRT/RT group 58.9 months (median 61.5 months, range 8–130 months) and for the LCS + chRT/RT group 63.2 months (median 63.0 months, range 18–120 months). A total of 76/129 (59%) patients with carcinoma of the larynx and hypopharynx responded to the questionnaire.

### Patient and tumour characteristics

A total of 42/76 (55.3%) patients, the majority of respondents, had a locally advanced tumour of UICC Stage IVA. The median age at diagnosis was 59 years (range 41–75 years). Laryngeal cancer was the main site of disease for 50/76 (66%) patients.

Patient characteristics are listed in Table 1. Due to the presence of a non-randomized study, there were some differences between the treatment groups. The defchRT/RT group showed a slightly higher proportion of poorer tumour and patient-specific characteristics in comparison with the patients treated with LE/LCS.

Dysphagia/hoarseness at the time of diagnosis has been reported in 4/17 (23.5%)/6/17 (35.3%) patients for the defchRT/RT group, 15/28 (53.6%)/21/28 (75%) patients for the LE + chRT/RT group, and 13/24 (54.2%)/6/24 (25%) patients for the LCS + chRT/RT group. In seven patients, no symptoms were documented at diagnosis.

### Voice/communication

The calculated average values for the communication component were 1.1 (range 0–3.2), 1.1 (range 0–2.5), and 0.9 (range 0–2.3) for the definitive chRT/RT, LE + chRT/RT, and LCS + chRT/RT groups, respectively (*P* = 0.6).

#### Hoarseness

Patients of the LE + chRT/RT group also stated that their voice sounded hoarse. Of these, five patients used a voice prosthesis, one an oesophageal substitute voice, and one an electrolarynx. A total of 14/21 (67%) patients of the defchRT/RT group felt that their voice sounded hoarse and, with a trend of significance, this was more often an issue of concern for this group in comparison with the 7/21 patients of the LE + chRT/RT group (67% vs 33%, *P* = 0.06).

There was also a trend to greater substantial–strong hoarseness after defchRT/RT treatment (10/21 patients) compared with 7/26 patients who underwent LCS + chRT/RT treatment (48% vs 27%, *P* = 0.2). For all other criteria, both treatment groups were equally satisfied.

#### Understandability in conversation and on the phone

The laryngectomees felt significantly more often misunderstood in conversation (*P* = 0.008) and on the phone (*P* < 0.05) compared with the LCS + chRT/RT group, indicating that the ability to communicate was worse after LE + chRT/RT. Members of the LE + chRT/RT group were also more frequently dependent on tools such as writing and gestures to communicate (*P* = 0.02) and demonstrated a trend towards greater dissatisfaction with the sound of their voice (*P* = 0.07).

#### Use of a speech aid

The analysis showed that 10/14 (71%) patients of the LE + chRT/RT group felt disturbed by their necessary speech aid. They also felt significantly more often misunderstood in conversation compared with the defchRT/RT group (83% vs 40%, *P* = 0.005). In comparison with the defchRT/RT group, there was a trend towards greater dissatisfaction with the sound of their voice (*P* = 0.3) and, additionally, patients felt that people in their environment perceived the sound of their voice as annoying (*P* = 0.09).

Tables 2 and 3 show the impact on communication/voice in the three treatment groups.

**Table 2.** Impact on communication/voice in the defchRT/RT group

Communication/Voice	defchRT/RT		Total patients
	Hardly–strong	Substantial–strong	
	*n* (%)	*n* (%)	*n* (%)
Hoarseness	14 (67)	10 (48)	21 (100)
Strenuous speaking	14 (70)	6 (30)	20 (100)
Annoying tone of voice	7 (35)	4 (20)	20 (100)
Tone of voice annoys people in environment	6 (35)	3 (18)	17 (100)
Tools to communicate (writing, gesture)	3 (15)	0 (0)	20 (100)
Misunderstood on the phone	8 (40)	5 (25)	20 (100)
Misunderstood in conversation	8 (40)	4 (20)	20 (100)
No communication possible	yes:	0 (0)	21 (100)
Use of speech aid	yes:	1 (5)	21 (100)
Speech aid annoying	0 (0)	0 (0)	1 (100)

Percentages refer to the patients who gave responses about the side effects. Specification ‘hardly–strong’ includes all answers made by the patients with ‘hardly’, ‘little’, ‘substantial’ or ‘strong’. defchRT/RT = definitive radiochemo-/radiotherapy.

**Table 3.** Impact on communication/voice in the LE + chRT/RT and LCS + chRT/RT groups

Communication/Voice	LE + chRT/RT		Total	LCS + chRT/RT		Total
	Hardly–strong	Substantial–strong		Hardly–strong	Substantial–strong	
	*n* (%)	*n* (%)	*n* (%)	*n* (%)	*n* (%)	*n* (%)
Hoarseness	7 (33)	4 (19)	21 (100)	14 (54)	7 (27)	26 (100)
Strenuous speaking	15 (68)	7 (32)	22 (100)	17 (65)	10 (38)	26 (100)
Annoying tone of voice	11 (52)	6 (29)	21 (100)	6 (23)	4 (15)	26 (100)
Tone of voice annoys people in environment	12 (67)	6 (33)	18 (100)	11 (42)	3 (11)	26 (100)
Tools to communicate (writing, gesture)	9 (36)	1 (4)	25 (100)	2 (7)	2 (7)	27 (100)
Misunderstood on the phone	16 (70)	3 (13)	23 (100)	10 (40)	3 (12)	25 (100)
Misunderstood in conversation	19 (83)	3 (13)	23 (100)	11 (44)	1 (4)	25 (100)
No communication possible	yes:	4 (15)	27 (100)	yes:	1 (4)	27 (100)
Use of speech aid	yes:	14 (54)	26 (100)	yes:	2 (8)	25 (100)
Speech aid annoying	10 (71)	3 (21)	14 (100)	0 (0)	0 (0)	2 (100)

Percentages refer to the patients who gave responses about the side effects. Specification ‘hardly–strong’ includes all answers made by the patients with ‘hardly’, ‘little’, ‘substantial’ or ‘strong’. LE + chRT/RT = laryngectomy + radiochemo-/radiotherapy, LCS + chRT/RT = larynx conservation surgery + radiochemo-/radiotherapy.

### Swallowing function

The calculated average values for the swallowing component were 0.8 (range 0–2.9), 0.5 (range 0–1.7) and 0.8 (range 0–2.3) for the defchRT/RT, LE + chRT/RT, and LCS + chRT/RT groups, respectively. Thus the laryngectomees demonstrated a trend towards fewer problems, but no significant group differences were noticed (*P* = 0.7).

Patients treated with defchRT/RT reported significantly greater difficulties with swallowing liquid food and substantial–strong difficulties with swallowing soft food in comparison with the LE + chRT/RT group (*P* < 0.05). Furthermore, greater substantial–strong problems with swallowing residuals that spill into the airway (as a possible clue to an existing aspiration) were stated compared with the LE + chRT/RT group (*P* < 0.05). In addition to the swallowing difficulties, 9/19 (47%) patients after defchRT/RT were dependent on a percutaneous endoscopic gastrostomy (PEG) feeding tube. Of these patients, seven used the PEG feeding tube in addition to normal diet, which was significantly more frequent in comparison with the LE + chRT/RT and LCS + chRT/RT groups (*P* < 0.05). Two patients were fully dependent on the PEG feeding tube for nutrition.

Additionally, patients treated with LCS + chRT/RT reported more frequent substantial–strong problems with swallowing residuals that spill into the airway compared with the LE + chRT/RT group (*P* < 0.05). They also reported greater swallowing difficulties with liquid food and an increased substantial–strong problem with burning oneself on the ingestion of food (*P* < 0.05).

Tables 4 and 5 indicate the effect on swallowing function in the three treatment groups, and Table 6 provides data on need for PEG-nutrition.

**Table 4.** Impact on swallowing function in the defchRT/RT group

Swallowing	defchRT/RT		Total patients
	Hardly–strong	Substantial–strong	
	*n* (%)	*n* (%)	*n* (%)
Solid food	12 (60)	9 (45)	20 (100)
Soft food	9 (45)	5 (25)	20 (100)
Liquid food	6 (30)	1 (5)	20 (100)
Granular food	10 (53)	8 (42)	19 (100)
Reduced gliding ability	11 (65)	8 (47)	17 (100)
Swallowing residuals	8 (47)	4 (23)	17 (100)
Pain	6 (35)	1 (6)	17 (100)
Avoid eating in society	5 (29)	4 (23)	17 (100)
Burning at eating	3 (18)	1 (6)	17 (100)

Percentages refer to the patients who gave responses about the side effects. Specification ‘hardly–strong’ includes all answers made by the patients with ‘hardly’, ‘little’, ‘substantial’ or ‘strong’. defchRT/RT = definitive radiochemo-/radiotherapy.

**Table 5.** Impact on swallowing function in the LE + chRT/RT and LCS + chRT/RT groups

Swallowing	LE + chRT/RT		Total	LCS + chRT/RT		Total
	Hardly–strong	Substantial–strong		Hardly–strong	Substantia–strong	
	*n* (%)	*n* (%)	*n* (%)	*n* (%)	*n* (%)	*n* (%)
Solid food	16 (61)	7 (27)	26 (100)	15 (58)	11 (42)	26 (100)
Soft food	5 (19)	0 (0)	26 (100)	8 (31)	1 (4)	26 (100)
Liquid food	1 (4)	1 (4)	26 (100)	10 (38)	1 (4)	26 (100)
Granular food	10 (43)	4 (17)	23 (100)	9 (39)	4 (17)	23 (100)
Reduced gliding ability	10 (50)	6 (30)	20 (100)	14 (70)	8 (40)	20 (100)
Swallowing residuals	7 (28)	0 (0)	25 (100)	17 (71)	4 (17)	24 (100)
Pain	6 (22)	0 (0)	27 (100)	9 (35)	0 (0)	26 (100)
Avoid eating in society	11 (44)	7 (28)	25 (100)	7 (27)	5 (19)	26 (100)
Burning at eating	6 (22)	0 (0)	27 (100)	7 (29)	4 (17)	24 (100)

Percentages refer to the patients who gave responses about the side effects. Specification ‘hardly–strong’ includes all answers made by the patients with ‘hardly’, ‘little’, ‘substantial’ or ‘strong’. LE + chRT/RT = laryngectomy + radiochemo-/radiotherapy, LCS + chRT/RT = larynx conservation surgery + radiochemo-/radiotherapy.

**Table 6.** Necessity of PEG feeding in the defchRT/RT, LE + chRT/RT and LCS + chRT/RT groups

PEG-feeding	None	Additional	Completely	Total
	*n* (%)	*n* (%)	*n* (%)	*n* (%)
defchRT/RT	10 (53)	7 (37)	2 (10)	19 (100)
LE + chRT/RT	25 (93)	1 (4)	1 (4)	27 (100)
LCS + chRT/RT	23 (85)	1 (4)	3 (11)	27 (100)

Percentages refer to the patients who gave responses about the side effects. defchRT/RT = definitive radiochemo-/radiotherapy, LE + chRT/RT = laryngectomy + radiochemo-/radiotherapy, LCS + chRT/RT larynx conservation surgery + radiochemo-/radiotherapy.

## DISCUSSION

Different therapy modalities for laryngeal and hypopharyngeal cancer are feasible. In several trials, at least since the results of randomized Phase 3 trial 24954 from the European Organization for Research and Treatment of Cancer (EORTC), induction chemotherapy followed by irradiation and concurrent chemotherapy and radiotherapy have been identified as comparable alternatives to total LE [3, 6, 27]. Nevertheless, it is not apparent that the best treatment modality has been established. Aside from the established factors (extent of the tumour, site of the tumour, lymph node involvement, and systemic metastasis), other important factors such as socioeconomic status, comorbidities, and refusal of tracheostomy are also meaningful in the process of deciding for or against a particular therapy modality [2, 28].

### Questionnaire

A comparative analysis of functional results is complex because of the fundamentally different therapeutic approaches. We are aware that the resulting vocal function after chemoradiotherapy or LCS is different from the estimated vocal function after LE with a substitute voice. One main concern of the present comparative analysis was to identify the impact on communicative ability caused by changes in vocal function, and the developed substitute voice induced by different therapy modalities.

It was important for us to evaluate in detail the impact experienced by patients in regard to communication/voice quality and swallowing function. For this reason, specific questionnaires were chosen to capture both the general situation and the functional results for swallowing and communication via in-depth and detailed questions [25, 26].

Due to poorer tumour characteristics in the defchRT/RT group, with a higher proportion of T4 tumours in comparison with the LCS + chRT/RT group and of N2 category in comparison with the LE + chRT/RT group, a worse outcome could be expected for the defchRT/RT group. The expected worse outcome for the defchRT/RT group might be the reason for the slightly shorter follow-up time in this group in comparison with the follow-up time of the other two groups. The treatment period of the analysed patients was between 1998 and 2007. Because of this long treatment period and the assumed better outcome for the LE + chRT/RT and LCS + chRT/RT groups due to favourable tumour characteristics, it could be expected that more patients would be alive after more than four years and could respond to the questionnaire in these two groups. However, because follow-up time in the defchRT/RT group was almost four years, meaningful conclusions on long-term toxicity were able to be obtained.

### Voice

Depending on the extent and site of the disease, patients may experience voice changes prior to therapy.

Several studies have described voice impairments after primary radio(chemo)therapy [15, 16, 29, 30]. Although patients in the study by Carrara *et al.* who underwent primary chemoradiation—the aim of which was organ preservation—suffered from a mild to moderate dysphonia, an understandable voice still remained [29]. In our analysis, patients after defchRT/RT reported very good voice function, or only occasionally occurring hoarseness. Additionally, patients after primary defchRT/RT reported (despite hoarseness) a better understandability of their speech compared with laryngectomees.

An important consideration in this context is follow-up care in the form of early voice therapy [31]. Unfortunately, valid data about the implementation and success rate of such care could not be determined for the present group of patients.

### Swallowing function

If swallowing function is already severely limited prior to therapy, then a LE is preferred. Vocal rehabilitation is therefore regarded as being secondary; the recovery of or backup for swallowing plays the central role.

Intensive chemoradiotherapy, however, is associated with increased side effects that can affect swallowing function. In our study, patients more frequently reported swallowing difficulties after defchRT/RT. For 7/19 (37%) patients, there was greater dependence on supplemental PEG feeding than experienced by patients after LE or LCS + chRT/RT. It was striking that 65% of the defchRT/RT patients reported a reduced gliding ability of food. This is consistent with the observation that the fibrosis of the oesophagus limits the structural function of the oesophagus and impairs swallowing [14, 17]. Likewise, a higher-grade xerostomia impairs the passage of food. Patients treated with LCS + chRT/RT also reported difficulties with swallowing in comparison with the LE + chRT/RT group. In this context they described a reduced gliding ability of food.

In considering the swallowing function after treatment, it should to be taken into account that patients in the present analysis were irradiated with older 2D- or 3D-planned radiotherapy techniques, mainly over laterally opposed fields. For the observation period, this was the radiotherapy technology in standard use. Various studies have found that impairment of swallowing correlates with the dosage of radiotherapy to structures involved in swallowing, especially the Musculi constrictores pharyngis and the glottic/supraglottic regions of the larynx [18, 19]. With the techniques used in the present evaluation, the average dose of radiation delivered to these structures was over 60 Gy, so a direct link with the high dysphagia rate can be assumed. Eisbruch *et al.* successfully reduced the volume exposed to more than 50 Gy (the optimization target) in the region of the Musculi constrictores pharyngis superior, medius and inferior by 10% on average with standard intensity-modulated radiation therapy (IMRT) compared with a 3D conformal technique, and achieved on average a further reduction of 10% with ‘dysphagia-optimized’ IMRT [19]. Recent calculations by Van der Laan *et al.* indicate it is possible to reduce dysphagia Grade 2–4 (RTOG) by 9% with a ‘swallowing-sparing’ IMRT compared with standard IMRT [32]. By using modern IMRT, which is nowadays becoming more and more the standard, it is possible to spare the anatomic structures that, if damaged, would cause dysphagia. Because of that a significant improvement in swallowing function can be expected.

It should also be taken into account that in our institute, prophylactic feeding tube (PEG) placement before radiotherapy is the standard approach. After completion of therapy, there are no fixed rules for the timely removal of the PEG tubes, which means that feeding tube duration might be longer than necessary. A non-randomized study comparing nasogastric tubes with PEG tubes in patients with head and neck cancer showed that patients with PEG tubes have a longer feeding tube duration, increased persistent dysphagia, and a greater need for pharyngoesophageal dilatation [33]. Thus the discomfort of the nasogastric tube motivates the patients to undergo oral intake of food, which effectively trains the swallowing muscles. However, patients with PEG tubes experience longer intervals of no oral intake and non-use of the swallowing musculature, perhaps leading to atrophy and persistent long-time dysphagia [34]. Therefore, there is controversy in the literature about the prophylactic PEG tube placement and uncertainty as to whether PEG or nasogastric tube feeding should be used [35].

The study has several limitations. It is retrospective and compares non-randomized groups of patients. The treatments in each group were not uniform. For example, the extent of the pharyngeal wall resection in the LE group was dependent on the extent and the site of the tumour, and the chemotherapy given in the definitive radio(chemo)therapy group varied. This may also affect post-therapy function. The number of patients was small, and due to the existence of a non-randomized collective, the treatment groups were heterogenic, making it difficult to arrive at significant conclusions. Furthermore, the questionnaire was only sent to the patients once, so a comparison between pre- and post-treatment function was not possible. Finally, the lack of questionnaire replies from all patients means that it was only possible to make restricted conclusions.

In summary, this retrospective analysis focussed on the impact of therapy on long-term perceived post-cancer-treatment function. Voice quality and communicative ability were slightly worse after defchRT/RT and LE + chRT/RT as a result of moderate impairments in comparison with LCS + chRT/RT, but were generally satisfying with all three treatment modalities. After defchRT/RT, dysphagia was more frequent. The reduction of chronic side effects in this sensitive region, therefore, remains a challenge for radiation oncology research, and measures of post-treatment function are important endpoints. To improve organ preservation and function, and thus long-term quality of life for patients, further development of the approach to therapy is necessary.
